# Transgenic solutions for the germline*

**DOI:** 10.1895/wormbook.1.148.1

**Published:** 2010-02-08

**Authors:** Christopher Merritt, Geraldine Seydoux

**Affiliations:** 1 Department of Molecular Biology and Genetics, Howard Hughes Medical Institute, and Center for Cell Dynamics, Johns Hopkins School of Medicine, 725 N. Wolfe St., PCTB 706, Baltimore, MD 21205, USA

## 1. Introduction

One of the most thrilling experiments in biology is to introduce a gene of one’s own design into a favorite animal and examine the effect in the transgenic progeny. Methods to construct, transform and monitor transgenes have been available to worm breeders since the pioneering work of Andy Fire and Craig Mello (Fire, 1986; Mello and Fire, 1995) and the introduction of green fluorescent protein (GFP) by Marty Chalfie (Chalfie et al., 1994). Sadly, for many years, the thrill of “seeing green” was denied to worm breeders working on the germline, as transgenes stubbornly refused to express in germ cells. In 1997, Bill Kelly and Andy Fire showed that transgene silencing in the germline is a copy-number driven process (Kelly et al., 1997). Multi-copy transgenes are expressed in the soma but silenced in the germline; in contrast, low-copy transgenes are expressed in both. Today, new transformation methods make it possible to routinely obtain low copy transgenes inserted in the genome. In this chapter, we review these methods and give practical advice for designing and transforming “germline-ready” transgenes.

## 2. The germline challenge

The experiments of Kelly and Fire showed that unlike somatic cells, germ cells are very efficient at silencing genes present in multi-copy. In some cases, multi-copy transgenes have also been observed to silence the corresponding endogenous locus and phenocopy a loss-of-function mutation (co-suppression) (Robert et al., 2004). Silencing affects transgene expression not only in all germ cells, but also in the blastomeres of early embryos, which depend primarily on maternal mRNAs and proteins synthesized during oogenesis.

Three methods have been described to obtain low-copy transgenes. The first, developed by Kelly and Fire, involve diluting the transgene with genomic DNA prior to injection to make a “complex array” (Kelly et al., 1997). Complex arrays, however, are difficult to obtain and prone to silencing over time. Two other methods yield transgenes that are inserted in the genome, either at random sites [gene-gun transformation (Praitis et al., 2001; Wilm et al., 1999)] or at a pre-selected site [Mos1-mediated Single Copy Insertion (MosSCI) (Frokjaer-Jensen et al., 2008)]. These methods yield stable lines that can express in the germline for many generations.

**Figure 1:**
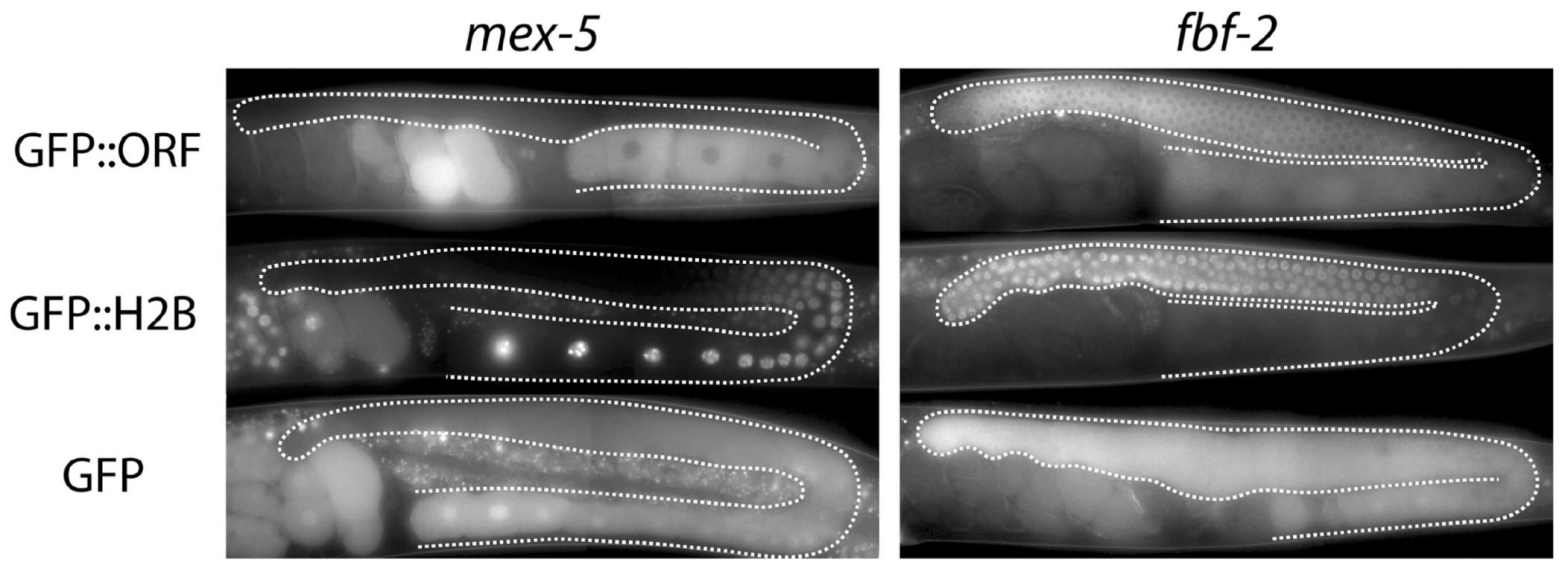
The choice of the ORF can affect the final observed pattern Comparison of three types of GFP fusions. Each fusion is driven by the *pie-1* promoter and the indicated 3′UTR (*mex-5* or *fbf-2*). GFP:ORF is an amino-terminal fusion between GFP and the indicated ORF (*mex-5* or *fbf-2*) (Merritt et al., 2008). GFP:H2B is an amino-terminal fusion between GFP and histone H2B. GFP is GFP alone. The GFP alone fusion gives the least accurate/specific pattern, due to diffusion through the rachis (C.M., *unpublished*). The GFP:H2B pattern reflects regulation by the 3′UTR only. The GFP:ORF pattern gives a pattern most similar to the endogenous pattern. Inclusion of the ORF is particularly important for the proper distribution of maternal proteins in embryos, which often depend on ORF sequences for segregation during early cleavages.

Besides silencing, gene expression in the germline presents two other unique challenges. First, unlike in the soma where promoters do the lion’s share of gene regulation, in the germline, 3′UTRs often (but not always) specify expression patterns (Merritt et al., 2008). Second, most of germline development occurs in a syncytium, where germ cells in different developmental stages are connected to a common central cytoplasm (rachis) (Hubbard and Greenstein, 2005). Diffusion into the rachis means that transgenic proteins, in particular small reporters like GFP, can end up far from the site of synthesis (Figure 1).

For these reasons, when designing germline transgenes, all components (promoter, tag, ORF and 3′UTR) must be considered carefully. Short cuts commonly used in somatic tissues, such as relying on simple promoter fusions (*promoter*::GFP::*unc-54 3′UTR*) to determine expression patterns (Hunt-Newbury et al., 2007), cannot be used in the germline. The methods presented in this chapter are designed to facilitate the cloning and mix-and-matching of different gene sequences to give the Worm Breeder maximum control over transgene design. The methods here are also compatible with the use of clones from publicly available collections, such as the promoterome (Dupuy et al., 2004), the ORFeome (Lamesch et al., 2004), and 3′UTRome (Mangone et al., 2008).

## 3. Transgene construction by modular cloning: the Multisite Gateway^®^ System

Modular cloning replaces traditional restriction-enzyme cloning with recombinational cloning, which allows users to rapidly recombine different parts (promoter, ORF and 3′UTR) of the transgene at will. Modular cloning is becoming more and more popular as collections of systematically-cloned promoters, ORFs, and 3′UTRs are becoming available. The method we describe in detail here is based on the Multisite Gateway^®^ System from Invitrogen. The Multisite Gateway^®^ system allows for rapid mix-and-match cloning of up to three DNA fragments in two steps:

Step 1: Construction of Entry Clones

**Figure 2:**
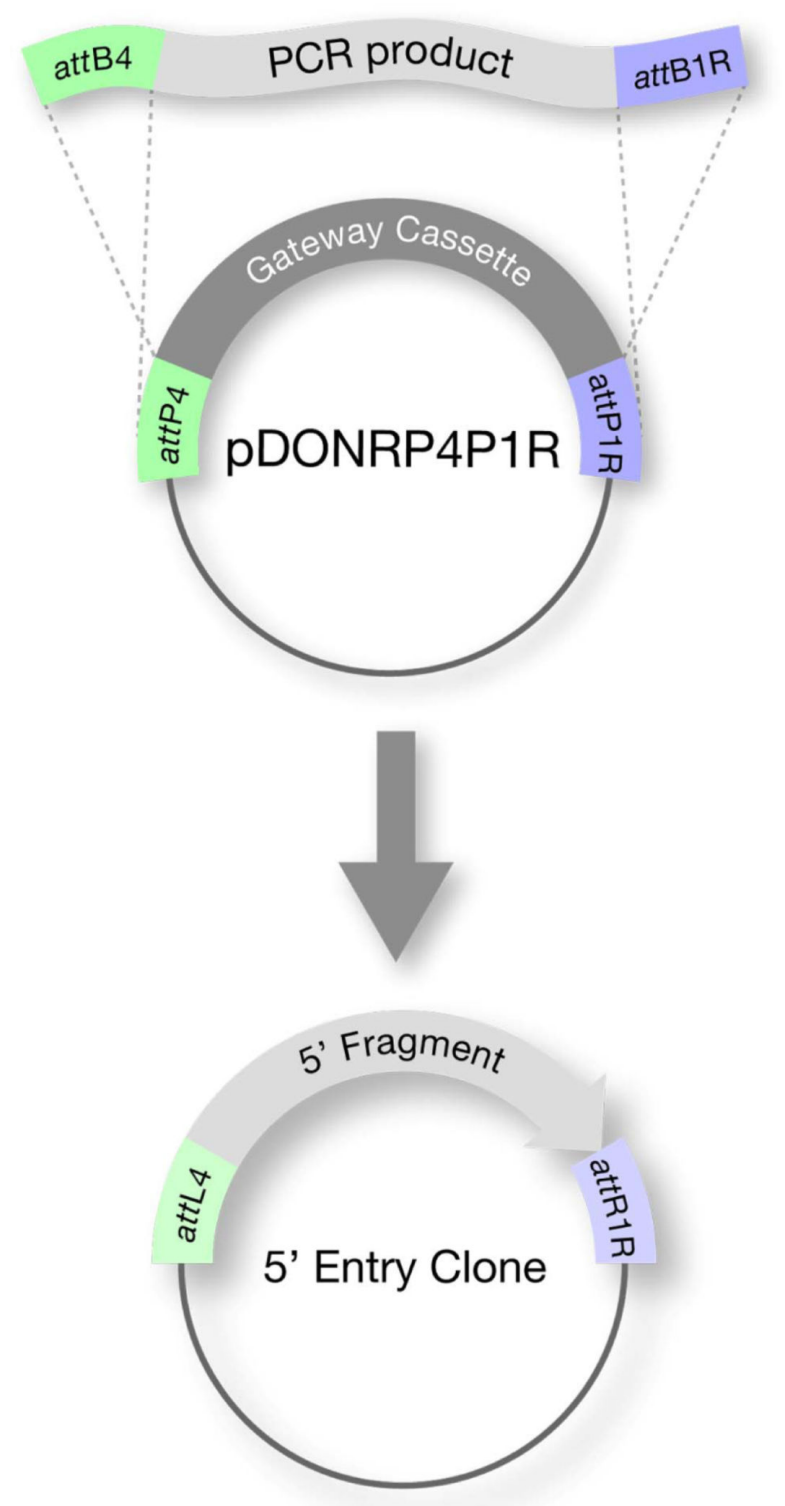
Step 1: Assembly of Entry Clone (BP reaction) The desired fragment is PCR amplified using oligos that contain *att*B sequences. Recombination with a Gateway^®^ Donor Vector gives rise to an Entry clone.

Each element (i.e. promoter, ORF and 3′ sequence) is cloned into a Donor Vector to create an Entry clone (Figure 2). There are three Donor Vectors, each corresponding to the specific position (5′, middle, or 3′) that the insert will occupy in the final clone (transformation-ready transgene). Typically promoters are cloned in the 5′ position, ORFs in the middle, and 3′UTRs in the 3′ position. Cloning is done by:

1. PCR amplification of the desired fragment using oligos that contain sequences complementary to the insert as well as sequences for cloning by recombination
2. recombination cloning (BP reaction) into one of the three Gateway^®^ Donor Vectors:
  5′ POSITION: pDONRP4-P1R, MIDDLE POSITION: pDONR201/221 (either can be used), 3′ POSITION: pDONRP2-P3
3. sequence verification of the resulting Entry clone to ensure that no mistakes were introduced during the PCR amplification.

Step 2: Assembly of transformation-ready transgene

**Figure 3:**
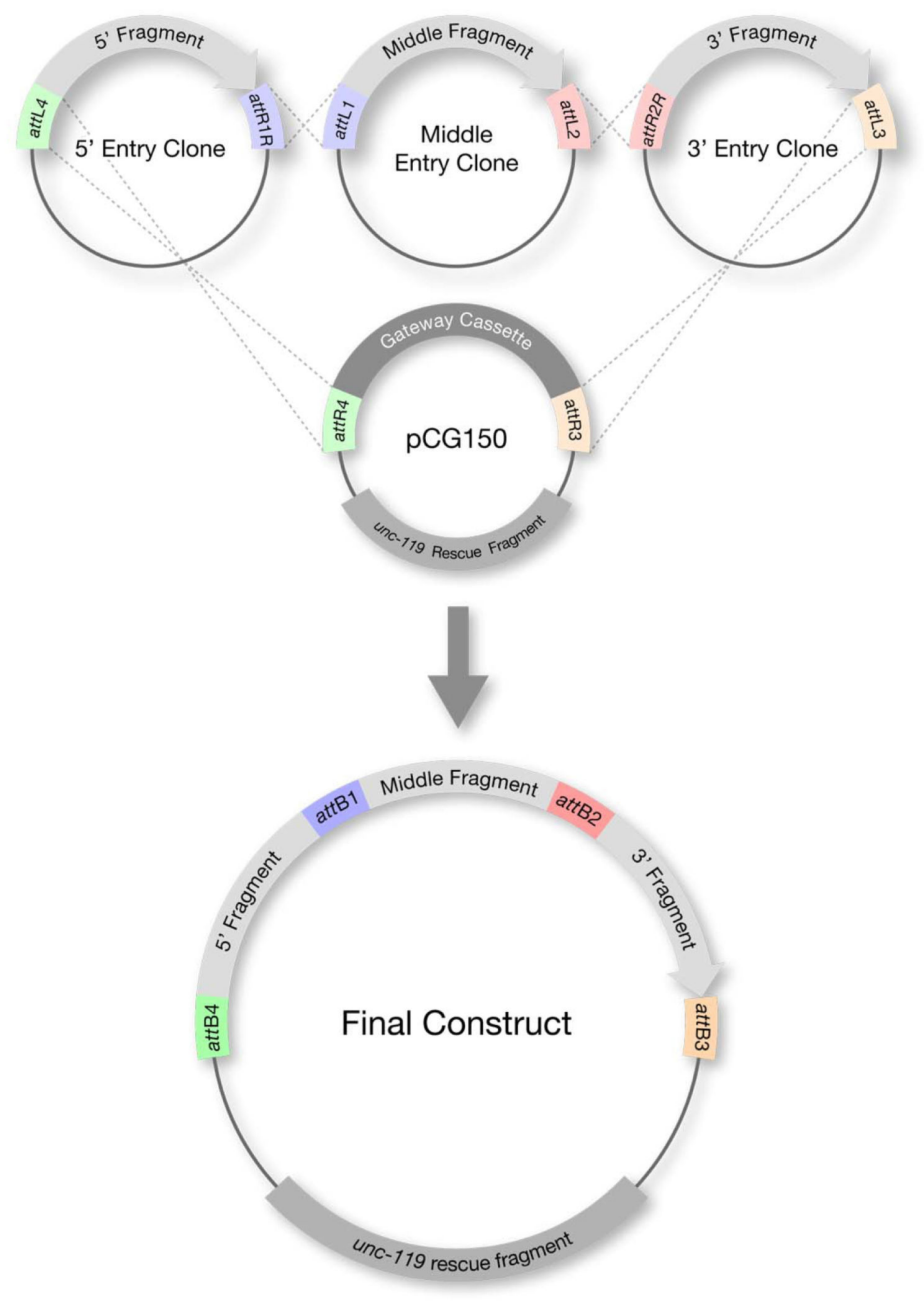
Step 2: Assembly of transformation-ready transgene (LR reaction) Three Entry clones are recombined in a single reaction with the Destination vector to give rise to the final construct (transformation-ready transgene). The Destination vector contains the marker used for transformation into worms (*unc-119* in this example).

Three Entry clones are recombined in a single cloning reaction (LR reaction) with the Destination vector (Figure 3). The Destination vector contains the marker(s) used for transformation into worms (for example: pCG150, which contains the *unc-119* rescuing fragment). The resulting final construct is the transgene ready for transformation.

## 4. Considerations when designing entry clones

For general instructions on how to design entry clones, please consult the Multisite Gateway^®^ Technology (Invitrogen) website (http://www.invitrogen.com/site/us/en/home/Products-and-Services/Applications/Cloning/Gateway-Cloning/MultiSite-Gateway-Technology.html) and manual (note: DO NOT use the Multisite Gateway^®^ “PRO” manual). See the Protocols section in this chapter for additional instructions/tips.

### 4.1. Choosing the promoter

- Promoter of gene of interest: this choice is recommended when the goal is to design a transgene that will most closely approximate the expression pattern of the gene of interest (in which case all parts of the gene (promoter, ORF and 3′UTR) must be used). Typically, the intergenic sequence from the end/start of the upstream ORF to the start codon of the gene of interest is sufficient. Large introns can also be included.

- *pie-1* promoter: this promoter is suitable for expression in all germ cells from the late L1 stage to the adult stage (Merritt et al., 2008). When combined with GFP:Histone H2B and tubulin 3′UTR, expression is first detected in the late L1 stage and is maintained in all germ cells through spermatogenesis and oogenesis. Maternal expression persists in embryos with declining levels after the 100-cell stage. More restricted expression can be obtained by combining the *pie-1* promoter with specific 3′UTRs, see below.

- *spe-11 promoter:* this promoter is active specifically during spermatogenesis in both hermaphrodites and males (Merritt et al., 2008). When combined with GFP:Histone H2B and tubulin 3′UTR, expression is first detected in late pachytene and is maintained through spermatogenesis.

- *hsp promoter:* for heat-inducible expression. CAUTION: These promoters are not germline-specific, they express in most somatic cells. In the germline, expression is first seen weakly in late pachytene of spermatogenesis and oogenesis (C. M. unpublished) several hours (usually, > 4 hrs) after heat-shock.

(See Table 1A for some useful 5′ Entry Clones containing promoters.)

### 4.2. Choosing the ORF

- ORF of gene of interest: this choice is recommended when the goal is to design a transgene that will most closely approximate the expression pattern of the gene of interest. Different proteins will have different turnover rates and diffusion patterns in the germline.

- GFP, mCherry, and other fluorescent proteins can be used in germ cells as has been described for somatic transgenes (see Reporter gene fusions). Note that since most germ cells are connected to each other through the rachis (with the exception of developing sperm), small proteins such as GFP can diffuse from cell to cell through most of the gonad (Figure 1).

- GFP:H2B (histone H2B) localizes efficiently to chromatin. This reporter is a good choice when the goal is to 1) concentrate the GFP signal to a small area to boost detection, 2) limit protein diffusion through the rachis, and 3) test the ability of a promoter or 3′UTR to restrict expression to specific stages (Merritt et al., 2008). Note that the rate of H2B turnover during germline development is not known and could in theory artificially extend or shorten an expression pattern.

(See Table 1B for some useful 5′ Entry Clones containing ORFs)

### 4.3. Choosing the 3′UTR

- 3′UTR of gene of interest: Many genes in the germline are regulated post-transcriptionally using sequences in the 3′UTRs (Merritt et al., 2008). Using the 3′UTR of the gene of interest is ESSENTIAL when the goal is to design a transgene that will most closely approximate the expression pattern of the gene of interest. See Table 1C and Table 2 for 3′UTRs with defined expression patterns.

- tbb-2 and unc-54 3′UTRs are compatible with expression in all germ cells (Merritt et al., 2008). See Tables 1C and 2 for some useful 3′ Entry Clones containing 3′UTRs.

### 4.4. Consideration when assembling a transformation-ready transgene

**Figure 4:**
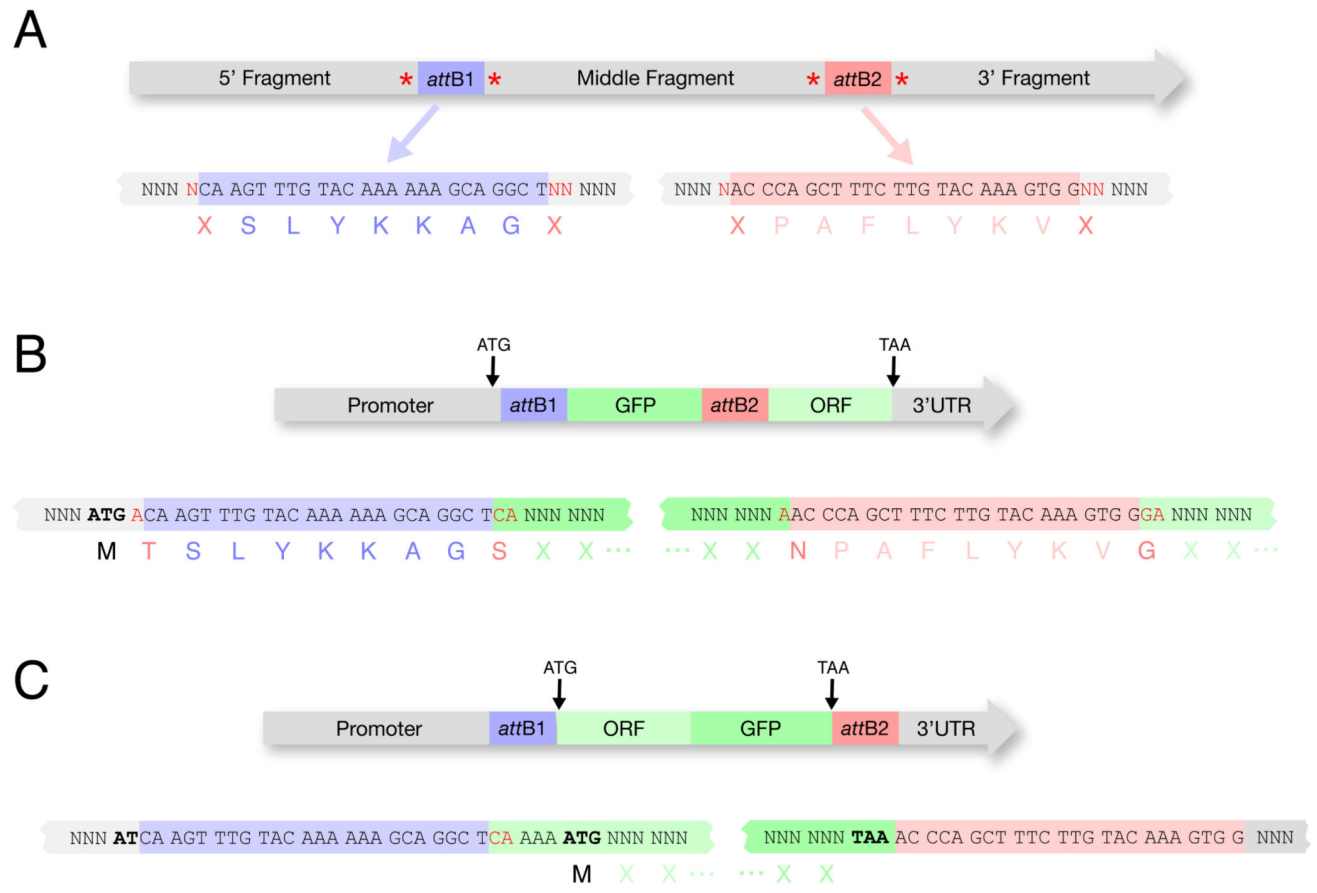
*att*B sites in final constructs A. The frame usage of *att*B sites must be considered for final constructs. These reading frames are suggested by Invitrogen and are used for all clones listed in Table 1. Extra nucleotides flanking the *att*B sites (in red) must be added for proper reading frame usage. B. Example of a construct with the start codon in the 5′ cassette and a stop codon in the 3′ cassette. In the 5′ cassette (containing a promoter) an A (in red) is added after the ATG so that the *att*B1 sequences will be in frame. In the middle cassette (containing GFP) CA (in red) is added 5′ of the GFP coding sequence and an A (in red) is added after the final GFP codon to maintain frame through the attB2 site. In the 3′ cassette (containing ORF::3′UTR) GA (in red) is added before the ORF coding sequence to maintain frame through the ORF. In this strategy, an available promoter can be used in the 5′ cassette (Table 1A) and an available fluorescent tag can be used in the middle cassette (Table 1B). The 3′ cassette containing ORF::3′UTR is the only entry clone that needs to be created by the researcher. We typically clone the ORF::3′UTR (with introns) directly from a genomic DNA template, but a cDNA template can also be used. C. Example of a construct with the start codon and stop codon in the middle cassette. This strategy avoids the inclusion of attB sequences in the final ORF. Available promoters (lacking ATG) (Table 1A) and 3′UTRs (Table 1C) can be used in the 5′ and 3′ cassettes. The middle cassette must contain the ORF and fluorescent tag fused by fusion PCR.

The main advantage of Gateway^®^ cloning is the ability to use pre-made Entry clones and thus reduce the number of clones that must be made *de novo* by the user. In general, the design of a transformation-ready transgene will be dictated by whatever pre-made Entry clones are available and the purpose of the transgene. For example, if the goal is simply to tag a protein under endogenous control, one could consider cloning the promoter in the first position, the tag in the second position, and the ORF + 3′UTR in the third position (amplified as a single unit from genomic DNA or cDNA) (Figure 4B). In this way, the user can take advantage of several pre-made middle Entry clones containing different tags (Table 1B) to rapidly create and test different tagged versions of the protein of interest.

### 4.5. Where to put the start and stop codon?

Placement of the start and stop codons will determine whether the final protein fusion will contain amino acids coded by the *att* recombination sites used in cloning. Ideally the start and stop codons should flank the ORF directly to avoid adding any extensions to the transgenic protein (Figure 4C). In practice though, if using clones from available libraries, you may be forced to consider a different strategy.

#### 4.5.1. - ATG in promoter

Some promoter entry clones contain ATGs (pCG142, pCM1.41, Vidal promoters), while others do not (pCM1.127, pCM1.128). If the ATG is included with the promoter in the first position clone, and the ORF is in the middle position, then 1) the ORF must be cloned so as to be in frame (this is typically done by adding 2 N’s to the 5′ end) and 2) the ORF will be extended at its 5′ end by 9 amino acids encoded by the attB site (Figure 4B).

#### 4.5.2. - STOP codon in ORF

If the STOP codon is included in the ORF (middle position) and the 3′UTR is in the third position, then the 3′UTR will be extended at its 5′ end by the *attB* site sequence. In our experience (Merritt et al., 2008), this is an acceptable compromise that does not appear to create problems.

#### 4.5.3. - STOP codon NOT in ORF

Some ORF clones (Vidal collection) do not contain STOP codons, in which case the STOP codon must be included with the 3′UTR. If the ORF is in the Middle position and the STOP+3′UTR are in the third position, the transgenic protein will be extended at its 3′ end by 9 amino acids encoded by the attB site.

### 4.6. Other Linker issue

**Figure 5:**
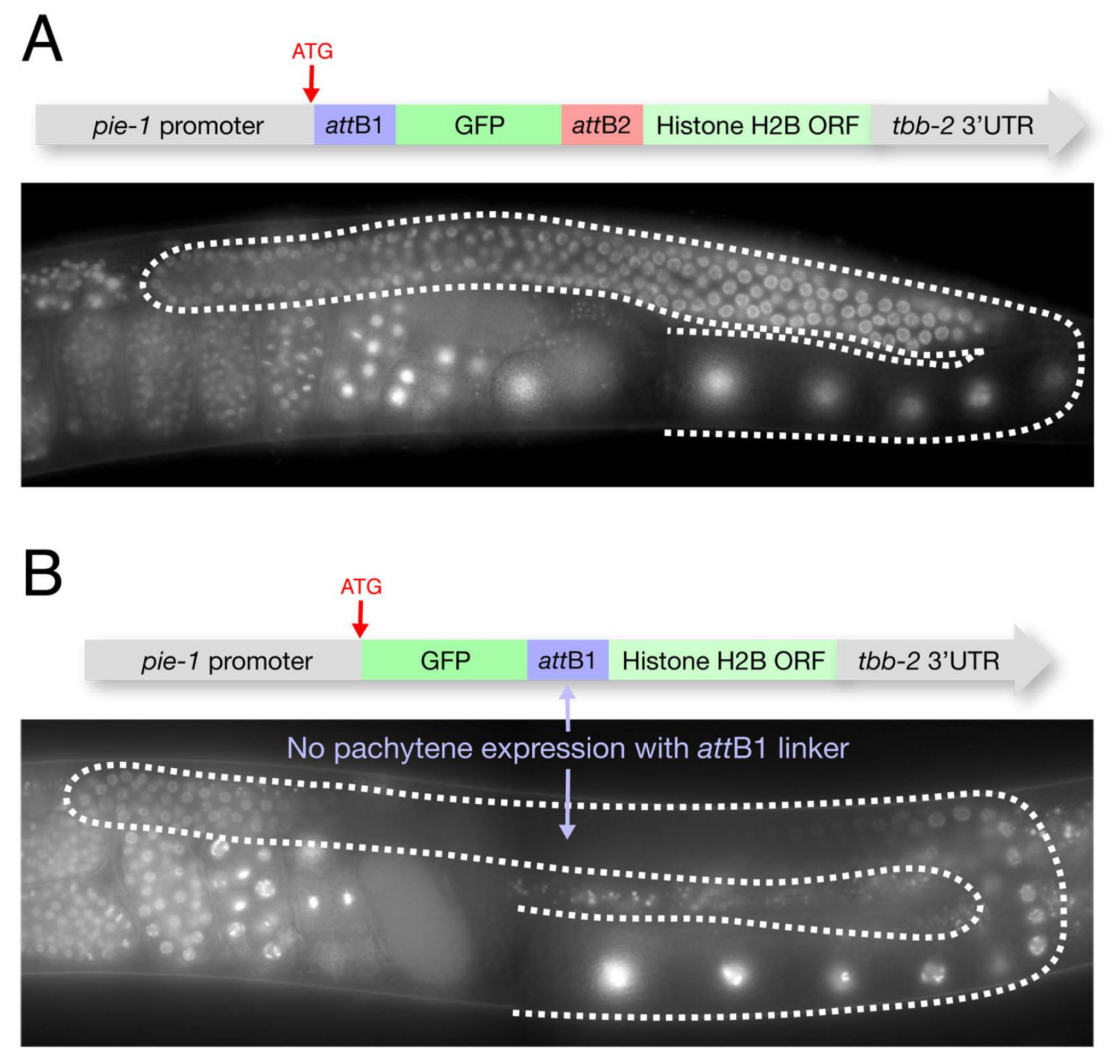
Inclusion of attB1 linker between GFP and ORF can affect distribution of fusion protein The *pie-1* promoter and *tbb-2* 3′UTR drive expression in all germ cells. However depending on the configuration of the *att*B sites, the fusion protein will behave differently. If the *att*B1 site is placed between GFP and H2B, the resulting fusion protein is unstable in pachytene nuclei (B). The *att*B2 site, however, does not cause this problem (A).

We have also found that placing the *att*B1 linker between GFP and ORF sequences can interfere with chromatin localization in germline pachytene nuclei. We have not observed this problem with the *attB2* linker (Figure 5).

## 5. Choosing a destination vector

Choice of destination vector will depend on the choice of transformation method to be used. There are currently two recommended transformation methods for germline transgenes: bombardment and Mos1-mediated Single Copy Insertion (MosSCI).

Bombardment does not require injection, but requires many worms (approximately 200,000 adults per construct) and is relatively slow ( 1 month), although hands-on time is low. Insertions are random throughout the genome. Expression levels are remarkably similar from line to line, and generally lower than endogenous levels (Seydoux lab, unpublished; Tenlen et al., 2008). Bombardment uses Destination Vector pCG150, which contains the *unc-119* minimal rescue fragment. See the bombardment protocol below for directions on how to use this marker effectively to obtain low copy insertions.

Mos1-mediated Single Copy Insertion (MosSCI) yields single-copy transgenes inserted at a pre-selected site. This method uses the standard injection method and does not require a gene gun. Stable lines can be obtained in less than 2 weeks. MosSCI uses Destination Vector PCFJ150. See Frokjaer-Jensen et al., 2008 and http://sites.google.com/site/jorgensenmossci/Home for protocols.

Destination Vectors MUST BE grown in DB3.1 bacteria. DB3.1 bacteria can carry plasmids with the *ccd*B “death gene” that is used as a negative selectable marker for Gateway^®^ cloning. Destination vectors should be selected on ampicillin (Amp) and chloramphenicol (Cm).

## 6. Non-Multisite Gateway^®^ vectors

### 6.1. Traditional cloning vectors

Table 4 shows commonly used vectors that use restriction enzyme cloning methods. These vectors contain *pie-1* promoter and *pie-1* 3′ sequences, and therefore are best suited for expression in oocytes and early embryos (maternal expression).

Other vectors only include a multiple cloning site and the transformation marker:

pDP#MM016b contains large genomic copy of the *unc-119*-rescuing fragment and pDP#MM051 contains a smaller copy of the *unc-119*-rescuing fragment (cDNA sequences instead of genomic sequences for the *unc-119* ORF) (Maduro and Pilgrim, 1995). In our hands, there is no difference in the rescuing ability of these *unc-119*-rescuing fragments.

pCFJ150 and pCFJ151 are two MCS-containing vectors that can be used for Mos1 mediated Single Copy Insertion (MosSCI) (Frokjaer-Jensen et al., 2008). These vectors contain the small *C. briggsae unc-119* rescuing fragment and an MCS flanked by Mos1 site sequences for insertion at Mos1 site by homologous recombination.

### 6.2. Single site Gateway^®^ vectors

Table 5 shows some commonly used single site Gateway^®^ vectors for embryonic expression. These vectors contain a Gateway^®^ cassette that is capable of accepting ORF sequences cloned into pDONR201/221 (equivalent to the middle Entry Clone in the Multisite Gateway^®^ system) These vectors all contain *pie-1* promoter and *pie-1* 3′ sequences to drive expression in oocytes and embryos.

## 7. Protocols

### 7.1. Gateway^®^ cloning

The following protocols are modified from Invitrogen’s protocols. Original protocols can be found on Invitrogen’s website (http://www.invitrogen.com/site/us/en/home.html).

### 7.2. Oligo design/PCR

To clone an insert into an Entry Vector, PCR primers must contain the flanking *att*B sites:

For pDONRP4-P1R (5′ Entry Clones) add:

*Forward - att*B4: 5′-GGGG ACA ACT TTG TAT AGA AAA GTT GNN

*Reverse - att*B1r: 5′-GGGG AC TGC TTT TTT GTA CAA ACT TGN

(where N’s put coding sequences in frame)

For pDONR201/221 (Middle Entry Clones) add:

*Forward - att*B1: 5′-GGGG ACA AGT TTG TAC AAA AAA GCA GGC TNN

*Reverse - att*B2: 5′-GGGG AC CAC TTT GTA CAA GAA AGC TGG GTN

(where N’s put coding sequences in frame)

For pDONRP2R-P3 (3′ Entry Clones) add:

*Forward - att*B2r: 5′-GGGG ACA GCT TTC TTG TAC AAA GTG GNN

Note: The MultiSite Gateway^®^ Three-Fragment Vector Construction Kit Manual, Version E, 16 August 2007 has the INCORRECT sequence of this oligo: GGGG ACA GCT TTC TTG TAT AGA AAA GTT GNN instead of the CORRECT sequence (above) that can be found in the manual Version G, 8 September 2008.

*Reverse- att*B3: 5′-GGGG AC AAC TTT GTA TAA TAA AGT TGN

(where N’s put coding sequences in frame)

Some examples:

1. To clone a promoter with its ATG into pDONRP4-P1R:
  Forward oligo - includes **attB4 (sense)**:: 5′ end of promoter sequence (sense): 5′-**GGGG ACA ACT TTG TAT AGA AAA GTT G** NNNNNN-3′
  Reverse oligo—includes **attB1r (antisense)**:: 3′ end of promoter sequence (antisense): 5′-**GGGG AC TGC TTT TTT GTA CAA ACT TG**T CAT NNNNNN-3′ (where CAT is the start codon (antisense of ATG), and T puts *att*B1r sequences in frame)
2. To clone a promoter without its ATG into pDONRP4-P1R:
  Forward oligo - includes **attB4 (sense)**:: 5′ end of promoter sequence (sense): 5′-**GGGG ACA ACT TTG TAT AGA AAA GTT G** NNNNNN-3′
  Reverse oligo—includes **attB1r (antisense)**:: 3′ end of promoter sequence (antisense): 5′-**GGGG AC TGC TTT TTT GTA CAA ACT TG**AT NNNNNN-3′ (where AT is the first two bases of the start codon (antisense))
3. To clone an ORF (incuding ATG and STOP codons) into pDONR201/221:
  Forward oligo - includes **attB1 (sense)**:: 5′ end of ORF sequence (sense): 5′-**GGGG ACA AGT TTG TAC AAA AAA GCA GGC T**CA ATG NNN-3′ (where CA puts *att*B1 sequences in frame, and ATG is the start codon)
    Note: We now clone all of ORFs with an additional AAA (Lys) codon upstream of the ATG. The oligos are designed in this manner (where AAA is the additional codon): 5′-**GGGG ACA AGT TTG TAC AAA AAA GCA GGC T**CA AAA ATG NNN-3′
    This is useful when using a promoter construct that doesn’t include an ATG (see Figure 3C) as the stretch of A’s helps in ATG selection (Marc Perry, personal communication). This construct can also be used with a promoter construct that has an ATG.
    Reverse oligo—includes **attB2(antisense)**:: 3′ end of ORF sequence (antisense): 5′-**GGGG AC CAC TTT GTA CAA GAA AGC TGG GT** TTA NNN-3′ (where TTA is the stop codon (antisense))
4. To clone a 3′UTR in to pDONRP2R-P3:
  Forward oligo - includes **attB2r (sense)**:: 5′ end of 3′UTR sequence (sense): 5′-**GGGG ACA GCT TTC TTG TAC AAA GTG G**GA TAA NNN-3′ (where GA puts the TAA stop codon in frame, creating a **G**GA (Ala) codon)
    Note: You need not include the stop codon if the ORF has one already. If the ORF has no stop codon (as in the Vidal ORFeome) including the STOP in the 3′UTR will add the attB2R sequence to your ORF.
    Reverse oligo—includes **attB3 (antisense)**:: 3′ end of 3′UTR sequence (antisense): 5′-**GGGG AC AAC TTT GTA TAA TAA AGT TG** NNNNNN-3′

A few notes about PCR:

- We typically PCR amplify with a high-fidelity polymerase (*i.e.* Phusion High Fidelity DNA Polymerase, Finnzymes). The template can be from any source (genomic DNA, cDNA, plasmid DNA, *etc.*).
- We typically perform a 50 μl PCR reaction and gel purify half of the reaction with the QIAquick Gel Extraction Kit (Qiagen). We typically elute the DNA from the column with 30 μl of H_2_ O and use 1-7 μl of this purified PCR for the BP reaction.

### 7.3. BP Reaction

1. For each reaction mix at RT in 1.5 mL tube:
  - gel purified PCR in H_2_ O ( 15-150 ng, or 1-7 μl of purified PCR - see above, add more PCR product for larger PCR inserts)
  - pDONR Vector ( 150 ng/μl) (typically add 1 μl of miniprepped pDONR vector)
  - TE (pH 8) to 8 μl
2. Thaw BP Clonase II enzyme mix on ice.
  Note: BP Clonase II enzyme mix includes the buffer!, the original BP Clonase enzyme mix (without the “II” in the name) did not contain the buffer and was added separately.
3. Add 2 μl of BP Clonase II enzyme mix (immediately return enzyme mix to freezer). Mix by vortexing, then microcentrifuge briefly.
4. Incubate at 25°C for 1 hour or overnight (we typically do overnight incubations for larger inserts or if there is very little PCR product)
5. Add 1 μl of the Proteinase K solution. Mix by vortexing, then microcentrifuge briefly.
6. Incubate samples at 37°C for 10 minutes.

### 7.4. BP Reaction Transformation

**Figure 6:**
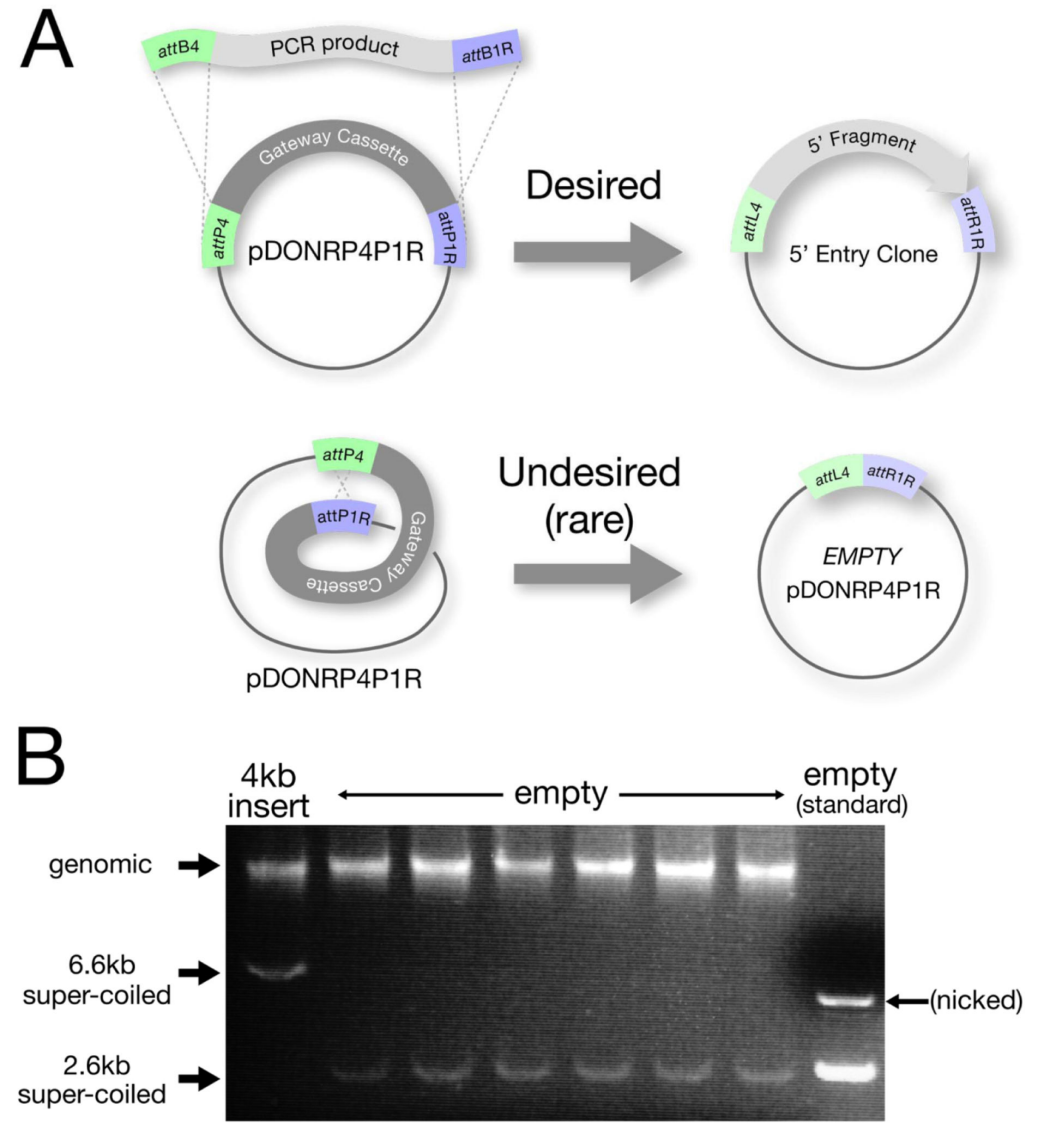
Troubleshooting the BP reaction when using large or low-yield PCR products A. Most BP reactions yield the desired Entry clone with insert. Empty pDONR plasmids resulting from an internal recombination reaction are obtained at a low frequency (C.M. unpublished) and are usually rare enough not to be a problem. Empty pDONRs can, however, become a nuisance (be more common) if the BP is performed with very small amounts of PCR insert or with a very long PCR insert ( 6 kb or greater). B. To help screen out empty pDONRs, DNA from a transformed colony is run directly on a gel to verify that the approximate size plasmid is present (for protocol see: “Rapid Screening by Direct Electrophoresis” section in Chapter 19 of E. coli Plasmid Vectors: Methods and Applications). Minipreps are then performed only on those colonies that contain the larger super-coiled plasmids. In this specific example, the BP reaction was done with a very low quantity (<15 ng) of a 4 kb PCR product. The colony on the far left contains the pDONR vector with the 4kb insert, while the next 6 colonies are all empty pDONRs.

1. Transform 2 μl of each BP reaction into 50 μl of Max Efficiency or Library Efficiency DH5 cells (DH5 cells with a transformation efficiency of 10^8^) (Subcloning Efficiency DH5 cells can be used for highly efficient BP reactions, i.e. very small inserts).
  Note: DH5 cells MUST be used for transformation. The success of the BP reaction depends on negative selection against the ccdB gene to select against “empty” pDONR vectors. Not all E. coli strains are sensitive to ccdB, for example DB3.1 the strain used to grow pDONR Vector, can tolerate ccdB. We have seen that XL1 Blue cells also tolerate ccdB.
2. Incubate on ice for 30 minutes.
3. Heat-shock cells at 42°C for 30 seconds.
4. Incubate on ice for 2 minutes.
5. Add 280 μl of S.O.C. Medium (2% tryptone, 0.5% yeast extract, 10 mM sodium chloride, 2.5 mM potassium chloride, 10 mM magnesium chloride, 10 mM magnesium sulfate, 20 mM glucose) and shake at 37°C for 1 hour.
  Note: use of S.O.C (available from Invitrogen), instead of LB, will result in many more colonies.
6. Plate 30 μl and 300 μl of each transformation onto Kanamycin plates (50 μg/ml) (NOT ampicillin!!)
7. Incubate at 37°C overnight.
8. Miniprep 2-4 colonies and roughly verify proper insert by restriction digest, PCR, or running of uncut DNA on gel (Figure 6). Confirm candidate preps by sequencing.

### 7.5. Multisite LR Reaction

1. For each reaction mix at RT in 1.5 ml tube:
  - 3 Entry Clones ( 10 fmoles each, typically just add 0.5 μl of miniprep)
  - Destination Vector ( 20 fmoles, typically just add 1 μl of miniprep)
  - TE (pH 8) to 8 μl
    Note: We typically do a negative control also - we remove one of the three Entry Clones from the reaction (and get 0 colonies)
2. Thaw LR Clonase II Plus enzyme mix on ice.
3. Add 2 μl of LR Clonase II Plus enzyme mix (immediately return enzyme mix to freezer). Mix by vortexing, then microcentrifuge briefly.
4. Incubate at 25°C overnight.
5. Add 1 μl of the Proteinase K solution. Mix by vortexing, then microcentrifuge briefly.
6. Incubate samples at 37°C for 10 minutes.

### 7.6. Multisite LR Reaction Transformation

1. Transform 2 μl of LR as described above for BP reaction BUT select on ampicillin (100 μg/ml) instead of Kanamycin!!
2. Miniprep 2-4 colonies and verify proper construct by restriction digest.

### 7.7. Bombardment Protocol

The bombardment technique can produce stable, low copy transgenes (1-3 copies, Praitis et al., 2001) that will not be silenced in the germline. Identification of transformed worms by bombardment is typically done by rescuing *unc-119* mutants with a wild-type copy of *unc-119* present on the bombardment plasmid. Bombardment has a very low frequency of successful transformation per adult bombarded, so a large number of worms must be bombarded (approximately 200,000 adults per construct). To select for transformed worms, worms are plated on several plates after bombardment and allowed to starve for several days. Over this time, the *unc-119*-rescued worms have a selective advantage over the non-transformed *unc-119* worms, which cannot form dauers and have difficulty surviving starvation (Praitis et al., 2001).

Several bombardment protocols have been published elsewhere (examples: Green et al., 2008; Praitis et al., 2001). The following protocol was created by the Seydoux lab and is adapted from Shai Shaham, personal communication, and Praitis et al., 2001. This protocol is for bombarding 3 different constructs.

Reagents:

Biolistic PDS-1000/He particle delivery system #165-2257 Bio-Rad
Hepta adapter, biolistic shock wave splitter #165-2225 Bio-Rad
Biolistic Microcarriers #165-2335 Bio-Rad
1 μM gold beads #165-2263 Bio-Rad
Rupture discs #165-2333 (1550psi) Bio-Rad
Hepta stopping screen #165-2226 Bio-Rad
Spermidine (tissue culture grade) S-4139 (5g) Sigma-Aldrich^®^
Nystatin N1638 (100 mls) Sigma-Aldrich^®^

You will need 160 large EP with Nystatin (NEP) plates spread with NA22 bacteria to bombard 3 constructs.

Enriched Peptone Plates with Nystatin (NEP) Recipe (1 Liter):

1.2 g sodium chloride
20 g peptone
25 g agar
H_2_ O to 1 liter
autoclave-cool to 55°C- then add STERILE:
1 ml cholesterol (5 mg/ml in EtOH)
1 ml 1 M MgSO4
25 ml 1 M potassium phosphate (pH 6.0)
10 ml Nystatin suspension (10,000 units/ml)

#### 7.7.1. Many days prior to bombardment: Growing up worms

##### 7.7.1.1. Master plates

Starting from an almost starved *unc-119 (ed3 or 4)* plate, take 10 piles of adult *unc-119* worms (*unc-119* worms form piles when starved) and place onto one small NGM plate with OP50 (see Maintenance of *C. elegans*, for NGM plate recipe). Repeat for one other NGM plate for a total of two plates. Let the worms grow at 25°C until food is gone and worms form piles.

##### 7.7.1.2. Amplify worms to 6 plates

Resuspend the 2 plates in 6 ml M9 buffer total. Spread 1 ml of supernatant to one large NEP plates with NA22 bacteria spread evenly over the entire plate. Repeat for a total of 6 plates. Let the worms grow at 25°C to starved L1s or nearly starved adults (see below).

Note: Worms can be grown at any temperature throughout the protocol (except after bombardment, where we recommend 25°C to encourage GFP expression). Grow at temperatures that are most convenient for you (from 15-25°C). The scheme above for initially growing up the worms does not have to be followed strictly. In the end, you just need several large plates (6 with newly starved L1s, or 12 with gravid adults).

#### 7.7.2. 2-4 days prior to bombardment: Final amplification of worms to 60 plates

The goal of the final amplification is to seed 60 plates with synchronized L1s (approx 20,000 L1s/plate) and grow them to fairly synchronized adults that can be bombarded.

##### 7.7.2.1. If starting with starved L1s

Resuspend each plate of freshly-starved L1 in 10 ml of M9 buffer (total 60 ml). Plate 1 ml of worm suspension onto a new large plate (60 total plates). Incubate the plates of L1s until they become young adults.

Note: USE ONLY FRESHLY-STARVED L1s. Plates that are over-starved will contain dead L1s and will yield fewer worms for bombardment. Drop a little M9 on the worm piles to make sure they contain healthy (wiggly) L1s.

If the plates contain many older worms as well as L1s, you must enrich for L1s by washing all of the plates off with M9 into a 50 mL conical tube and letting the worms sit on the bench for about a minute, letting the large worms sink to the bottom. Pipet the supernatant (enriched with L1s) into a new 50mL conical and use these to spread to 60 plates.

##### 7.7.2.2. If starting with nearly starved (gravid) adults

Collect adults in M9, precipitate and resuspend in bleach mixture (1 part 10N NaOH, 1 part Chlorox bleach, 8 parts M9). Rock worms for 7 minutes, spin, wash in M9. Amplify each plate of bleached eggs to 10 plates (total of 60 plates). Incubate the plates until worms become young adults.

Note: For best synchronization, eggs can be hatched overnight in M9 before plating on the 60 plates. If worms are hatched in M9 overnight, it is easy to count L1s. Ideally, 20,000 L1 should be plated per large NEP plate. With this number of worms, the plates will not starve completely before the worms reach adulthood, but will have low enough food to facilitate washing off the worms. Typically, 6 plates of adults with eggs are sufficient to collect enough L1s for 60 plates. To be safe, we often bleach more that 6 plates (12+ plates) to ensure that we obtain enough L1s for bombardment.

#### 7.7.3. Bombardment Day

Autoclave Hepta adaptor, microcarrier holder, microcarriers, mesh stopping screens and forceps.

##### 7.7.3.1. Worm preparation

Make sure most worms are YOUNG ADULTS (hermaphrodites with single row of embryos).

Wash worms from the 60 plates with M9 into a 50 ml tube. WATCH FOR CONTAMINATION. Spin at low speed for 1 minute at room temperature; wash worms until M9 solution becomes clear and finally transfer worms to a 15 ml tube. Spin at low speed, you should end up with a 3-4 ml pellet of packed adult worms. Remove liquid and resuspend worms in M9 to a total volume of 12 ml.

Using a Pasteur pipette, spread worms onto the surface of a dry enriched peptone plate with NA22. Add them drop-wise starting at the center and then spiraling around until you reach the edge of the plate. Repeat until all worms are plated (6 plates).

Leave the covers off the plates to evaporate the liquid. This should take no more than 15 min.

While the plates dry, prepare the gene gun. We use the Bio-Rad Biolistic PDS-1000/He particle delivery system with Hepta adaptor. This adaptor saves an enormous amount of time and effort. You can find the illustrations and definitions of the various jargon terms (microcarriers and microcarrier holder) in the Bio-Rad manual. Read this manual to be familiar with the procedures described below.

Wipe down the bombardment chamber and chamber door with 70% EtOH. Unscrew the helium pressure gauge and immerse in isopropanol for 15 min. or longer. Screw back the helium pressure gauge.

##### 7.7.3.2. DNA preparation

Weigh 35—50 mg of 1 μM gold beads (Bio-Rad) into siliconized 1.5 ml eppendorf tube.

Note: tungsten beads have also been used successfully (Jadhav et al., 2008)

Add 1 ml 70% EtOH. Vortex 5min. Soak for 15 min. Pellet and remove supernatant
Add 1 ml sterile water. Vortex 1min. Soak for 1 min. Pellet and remove supernatant
Add 1 ml sterile water. Vortex 1min. Soak for 1 min. Pellet and remove supernatant
Add 1 ml sterile water. Vortex 1min. Soak for 1 min. Pellet and remove supernatant

Resuspend in 500 μl sterile 50% glycerol. This bead stock can be used for 2 weeks (or more and should be stored at 4°C).

Vortex mix for 5min. Remove immediately 100 μl of bead suspension and place into a siliconized eppendorf tube. Repeat twice for a total of 3 tubes. Make sure to keep the gold beads in suspension. One tube will be used for each construct.

To each tube, add in order while vortexing on medium speed:

10 μg DNA (typically, 30-50 μl of miniprep, or 10 μl of 1 μg/μl midi)
100 μl 2.5M CaCl_2_
40 μl 0.1M spermidine

Vortex 2min. Soak for 1min. Pellet and remove supernatant.

Add 280 μl 70%EtOH. Flick tube to mix. Pellet and remove supernatant.
Add 280 μl 100%EtOH. Flick tube to mix. Pellet and remove sup. Add 100 μl 100%EtOH and resuspend by gently flicking tube. This is your prepped DNA.

##### 7.7.3.3. Bombardment

Open vacuum port, turn on gene gun. Open helium tank valve (check to make sure the He tank pressure is >2200psi). Close door.

1. Vortex your DNA preparation at medium speed with cap closed. Stop and quickly transfer 7 μl of beads onto the middle of a microcarrier. Repeat for 14 microcarriers total. Let ethanol evaporate (this only takes a few minutes).
2. Place 7 microcarriers (with dried DNA-gold beads) onto the Hepta adaptor microcarrier holder using forceps and tighten up with the special tool.
3. Place a rupture disk soaked in isopropanol in the retaining cap and tighten. Place stopping screen and microcarrier holder in chamber as described in the manual. Place uncovered worm plate (taped to the sample holder using a rolled piece of adhesive tape to create double sided tape) onto second rung in bombardment chamber.
4. Pull vacuum to 27 In. of Hg. Press Fire button until disk ruptures. Release vacuum (Vent position), and remove plate.
5. Repeat 1-4 for with the next 7 microcarriers and a new plate of worms.
6. Repeat 1-5 for the next two DNA preparations (each construct is bombarded onto two plates)
7. Turn off vacuum. Close helium tank valve. Make sure no pressure is left in the line. Turn the gene gun power OFF.

##### 7.7.3.4. Plating worms

Resuspend each plate of bombarded worms in 13 ml of M9 (repeat for each of the 6 plates). Plate 1 ml of worms onto each fresh NEP plate (approximately 8,000 bombarded worms per plate). For each construct, you will have 26 plates or 78 plates for all three constructs.

Note: To reduce contamination at this step, add 2.5 μg/ml Fungizone (amphotericin B) and 20 μg/ml tetracycline to the M9 to inhibit fungal and bacterial growth.

Let plates dry on the bench and incubate at 25°C for 2 weeks. The worms should starve no sooner than Day 3 (if kept at 25°C). If they starve earlier, you may not obtain stable integrants. It is very important to plate the bombarded worms at a low enough density to avoid starvation in the first 3 days.

#### 7.7.4. 2 weeks after bombardment

Scan plates for wild-type worms.

Note: You can continue to scan for wild-type worms for 2 additional weeks.

From each plate with wild-type worms, pick 3-5 worms and transfer them to one small NGM plate. Place the plates at 25°C for 4 days, and then examine the progeny for GFP expression. You can clone out at this point (pick a single GFP positive worm from one plate to start the line). Make sure the lines are stable (do not segregate Uncs). Lines that segregate Uncs tend to loose germline expression over time. Keep the lines at 25°C to maintain GFP (not all lines need to be kept at 25°C but many do).

In our hands, GFP expression in the germline is observed in 10-50% of independent *unc-119* rescued lines (lines are considered “independent” when isolated from different plates). We typically bombard the same transgene twice, on different days, to increase the likelihood of getting a line that expresses in the germline (transformation success can vary from day to day). If 20 or more *unc-119* rescued lines (stable) are obtained and none express in the germline, it may be helpful to bombard in parallel a construct known to express well (examples: pCM1.34 (*pie-1* promoter::GFP::histone H2B::*tbb-2* 3′UTR) or pCM4.09 (*pie-1* promoter::GFP::*pie-1* ORF::*pie-1* 3′UTR (Merritt et al., 2008) to verify the technique is working.

Final note: In the end, there are several key steps to a successful bombardment:

1. Start with synchronized worms
2. Bombard adults with no more than 1 row of eggs
3. Bombard 0.5mL of packed adults per plate
4. After bombardment, plate worms onto new plates so they starve in 3 days, NOT EARLIER!
5. Avoid contamination!!
6. Keep lines at 25°C.

## Figures and Tables

**Table 1:** Selected Entry Clones

A. 5′ Entry Clones				
Element	Application (to drive expression)	Description	Entry Clone (reference)	Distributed by
*pie-1* promoter	throughout the germline	Contains promoter + large intron from *pie-1* locus (3.0kb total in length)	pCG142 (Merritt et al., 2008)	Addgene (http://www.addgene.org/pgvec1)
*pie-1* promoter (short)	throughout the germline	smaller *pie-1* promoter (1.1 kb) lacking repetitive sequences (which are thought to be problematic with MosSCI)	pCM1.58 (C.M. unpublished)	Addgene (http://www.addgene.org/pgvec1)
*pie-1* promoter (short, no ATG)	throughout the germline	smaller *pie-1* promoter (1.1 kb) with no start codon. Avoids attB1-encoded amino-acids in fusion protein. ATG must be included in middle cassette (see Figure 4C).	pCM1.127 (C.M. unpublished)	Addgene (http://www.addgene.org/pgvec1)
*spe-11* promoter	in sperm	272 bp *spe-11* promoter.	pCM1.41 (Merritt et al., 2008)	Addgene (http://www.addgene.org/pgvec1)
*spe-11* promoter (no ATG)	in sperm	272 bp *spe-11* promoter with no start codon. Avoids attB1-encoded AAs in final fusion protein. ATG must be included in middle cassette (see Figure 4C).	pCM1.128 (C.M. unpublished)	Addgene (http://www.addgene.org/pgvec1)
hsp16-2/hsp16-41	heat-inducible	heatshock promoters commonly used for heat-inducible expression. Cloned from vectors created by Andy Fire.	pCM1.56/1.57 (C.M. unpublished)	Addgene (http://www.addgene.org/pgvec1)
Other germline promoters (Seydoux lab)	throughout the germline	many other germline promoters ranging in length from 400bp to 3000bp (see Merritt et al., 2008 - Table S5).	various (Merritt et al., 2008)	Addgene (http://www.addgene.org/pgvec1)
*C. elegans* Promoterome library (Vidal lab)	specific (somatic) cell-types	These clones contain up to 2 kb of intergenic sequence upstream of the ATG. They contain ATGs and are cloned as shown in Figure 4B.	various (Dupuy et al., 2004)	Geneservice (http://www.geneservice.co.uk/products/clones/Celegans_Prom.jsp)

B. Middle Entry Clones				
Element	Application	Description	Entry Clone (REF)	Distributed by
GFP	tag ORF with GFP	GFP with 3 synthetic introns (from Andy Fire)	pCM1.53 (Merritt et al., 2008)	Addgene (http://www.addgene.org/pgvec1)
mCherry	tag ORF with mCherry	mCherry with *C*. *elegans* codon preferences and 3 synthetic introns (created by Chris Gallo; Seydoux lab)	pCG144 (Merritt et al., 2008)	Addgene (http://www.addgene.org/pgvec1)
GFP:histone H2B	reporter		pCM1.35 (Merritt et al., 2008)	Addgene (http://www.addgene.org/pgvec1)
mCherry:histone H2B	reporter		pCM1.151 (Merritt et al., 2008)	Addgene (http://www.addgene.org/pgvec1)
*C. elegans* ORFeome library (Vidal lab)	express ORF	these ORFs do not contain start or stop codons and thus must be added in flanking cassettes	various (http://worfdb.dfci.harvard.edu; Dupuy et al., 2004)	Geneservice (http://www.geneservice.co.uk/products/cdna/Celegans_ORF.jsp)

C. 3′ Entry Clones				
Element	Application (to drive expression)	Description	Entry Clone (REF)	Distributed by
*tbb-2 3′UTR*	in all cell types	3′UTR permissive for expression throughout the germline	pCM1.36 (Merritt et al., 2008)	Addgene (http://www.addgene.org/pgvec1)
*pie-1 3′UTR*	in oocytes and embryos	3′UTR with highest expression in oocytes and early embryos	pCM5.47 (Merritt et al., 2008)	Addgene (http://www.addgene.org/pgvec1)
*unc-54 3′UTR*	in all cell types	3′UTR permissive for expression in all cells (Fire vectors)	pCM5.37 (Merritt et al., 2008)	Addgene (http://www.addgene.org/pgvec1)
*C. elegans* UTRome library (Piano lab)	to drive expression in various cell types	Last 30 bases of ORF sequence + STOP codon + 3′UTR sequence to polyA	various (UTRome.org; Mangone et al., 2008))	*not yet determined*
Other germline 3′UTRs (Seydoux lab)	to drive expression in different germline cell types		various (see Table 2) (Merritt et al., 2008)	Addgene (http://www.addgene.org/pgvec1)

**Table 2:** 3′UTRs with different expression patterns in the germline

		progenitors		pachytene		loop	oocytes			embryos (C.M. unpublished)	
3′UTR	Entry	distal	proximal	distal	proximal		distal	medial	proximal	early	late
*tbb-2* ^1^	pCM1.36	+	+	++	++	++	++	++	++	++	++
*pgl-1*	pCM5.30	++	++	++	++	+	+	+	++	++	++
*nos-3*	pCM5.35	++	++	++	++	+	+	+	+	++	++
*glp-1*	pCM5.40	++	++	++	++	+	+	+	+	+	++
*fbf-1* ^2^	pCM5.33	++	++	+	+	+					+ (posterior)
*daz-1*	pCM5.43	+	+	++	++	+	+	+	+	+	+
*pgl-3*	pCM5.46	+	+	+	++	++	++	++	++	++	++
*gld-1*	pCM5.36		+	++	+	+	+	+	+		+ (posterior)
*him-3*	pCM5.52			++	++	++	+	+	+	+	+
*rme-2* ^3^	pCM5.51			+	++	++	++	++	++	++	++
*mex-5*	pCM5.48				++	++	++	++	++	++	++
*puf-5*	pCM5.64				++	++	++	++	++	++	++
*pal-1*	pCM5.49				++	++	++	++	++	+	++
*spn-4*	pCM5.50	+	+						++	++	++
*pie-1*	pCM5.47	+	+	+	+	+	+	++	++	++	++
*pos-1*	pCM5.66	+	+	+	+	+	+	+	++	++	++
*cye-1* ^4^	pCM5.54	++	++	+	+	++	++	++	++	++	++
*cep-1*	pCM5.44	++	++	+	+	++	++	++	++	+	+
*mex-3*	pCM5.45	+	+			+	+	+	++	++	++
*fog-1*	pCM5.60		++	+	++	+	+	+	+	+	+

Germline expression of GFP:H2B when driven by the given 3′UTR (Merritt et al., 2008). Gonadal regions are listed distally to proximally, from left to right. ++ indicates strongest domain(s) of GFP:H2B expression, + indicates weaker domain(s) of GFP expression, and − indicates no GFP expression. Other 3′UTRs that provide similar expression patterns:

1 mes-2, par-5, fog-2, spe-11, spe-38, spe-41,

2 fbf-2,

3 lip-1, and

4 *mes-3*.

**Table 3:** Selected Destination Vectors

Destination Vector (reference)	Selectable Marker	Description	Distributed by
pDESTR4-R3	*none*	original Destination Vector for Multisite Gateway^®^ cloning	Invitrogen
pCG150 (Merritt et al., 2008)	*unc-119*	pDESTR4-R3 Destination Vector with a 2.2 kb *C*. *elegans unc-119* rescuing fragment (with cDNA as ORF) cloned into the vector backbone.	Addgene (http://www.addgene.org/pgvec1)
pCFJ150(Frøkjaer-Jensen et al., 2008)	*unc-119*	MosSCI Destination Vector. Contains 2.1 kb *C*. *briggsae unc-119* rescuing fragment and Multisite Gateway^®^ cassette flanked by Mos1 site sequences for insertion at Mos1 site by homologous recombination.	Addgene (http://www.addgene.org/pgvec1)

**Table 4:** Traditional cloning vectors for embryonic expression

Vector (reference)	Selectable Marker	Vector components	Description	Distributed by
pJH4.52 (Strome et al., 2001)	*none*	*pie-1* promoter::GFP:: (**SpeI**) **H2B (SpeI**)::*pie-1* 3′	Replace Histone H2B with ORF of interest using flanking SpeI sites	Geraldine Seydoux gseydoux@jhmi.edu
pAZ132 (Praitis, et al 2001)	*unc-119*	*pie-1* promoter::GFP:: (**SpeI**) **H2B (SpeI**)::*pie-1* 3′	Replace Histone H2B with ORF of interest using flanking SpeI sites	Vida Praitis praitis@grinnell.edu
pIC26 (http://jura.wi.mit.edu/cheeseman/Plasmids.php)	*unc-119*	*pie-1* promoter::GFP::TEV:: S-peptide::(**SpeI**):: *pie-1* 3′	Worm LAP construct (with GFP), clone ORF of interest into SpeI site	Iain Cheeseman icheese@wi.mit.edu
pAA65 (http://jura.wi.mit.edu/cheeseman/Plasmids.php)	*unc-119*	*pie-1* promoter::mCherry:: TEV::S-peptide:: (**SpeI**):: *pie-1* 3′	Worm LAP construct (with mCherry), clone ORF of interest into SpeI site	Iain Cheeseman icheese@wi.mit.edu
pSO26 (O’Rourke et al., 2007)	*unc-119*	*pie-1* promoter::GFP::TEV:: S-peptide::**MCS(6)** ::*pie-1* 3′	Worm LAP construct (with GFP) derived from pIC26 (above), contains 6 restriction sites (MCS) in place of SpeI.	Bruce Bowerman bbowerman@molbio.uoregon.edu

**Table 5:** 

Vector (reference)	Selectable Marker	Vector components	Description	Distributed by
pKR2.40 (Reese et al., 2000)	*none*	*pie-1* promoter::GFP::[**Gate** **way^®^ Cassette**] ::*pie-1* 3′	To GFP tag ORF, does not contain *unc-119* rescuing fragment	Geraldine Seydoux gseydoux@jhmi.edu
pID2.02 (D’Agostino et al., 2006)	*unc-119*	*pie-1* promoter::[**Gate** **way^®^ Cassette**] ::*pie-1* 3′	To drive ORF, ORF must be fused with tag by researcher if desired	Geraldine Seydoux gseydoux@jhmi.edu
pID3.01 (D’Agostino et al., 2006)	*unc-119*	*pie-1* promoter::GFP::[**Gate** **way^®^ Cassette**] ::*pie-1* 3′	To GFP tag ORF	Geraldine Seydoux gseydoux@jhmi.edu
pCM2.03 (Merritt et al., 2008)	*unc-119*	*pie-1* promoter::GFP::[**Gate** **way^®^ Cassette**]	To GFP tag ORF under the control of any 3′UTR. 3′UTR sequences must also be included downstream of ORF.	Geraldine Seydoux gseydoux@jhmi.edu
